# Enhanced Commendable Sensitivity and Specificity for MSI in Colorectal Cancer by a New PCR‐HRM Based 8‐Loci MSI Assay

**DOI:** 10.1002/jcla.25085

**Published:** 2024-08-12

**Authors:** Xueping Xiang, Xiaojing Ma, Linlin Ying, Hong Zou

**Affiliations:** ^1^ Department of Pathology, The Second Affiliated Hospital, School of Medicine Zhejiang University Hangzhou Zhejiang China

**Keywords:** colorectal cancer, diagnostic, immunohistochemistry, microsatellite instability, mismatch repair

## Abstract

**Background:**

This study evaluated the performance of the PCR‐HRM assay by comparing it with immunohistochemistry (IHC) for mismatch repair (MMR) proteins and the PCR capillary electrophoresis (PCR‐CE) methods.

**Results:**

A total of 224 patients with colorectal cancer participated in the study, with nearly half having mismatch repair deficiency (dMMR) tissues and the remainder possessing pMMR tissues. There was a 97.77% concordance between the PCR‐HRM assay and IHC, and a 97.56% concordance between PCR‐HRM and the PCR‐CE assay. In comparison with IHC for dMMR proteins, the PCR‐HRM demonstrated a sensitivity of 96.36% and a specificity of 99.12%. When juxtaposed with the PCR‐CE assay, its sensitivity was 98.96% and specificity stood at 96.33%. The mutations observed in the microsatellite loci were uniformly distributed across all eight loci. Discrepant outcomes were more frequent in instances of MLH1 and PMS2 deficiency. Furthermore, the germline mutation status of MLH1, MSH2, PMS2, and MSH6 in 62 patients was ascertained using next‐generation sequencing. All patients displaying MMR gene pathogenic mutations (*N* = 14) were identified as MSI‐H by PCR‐HRM, whereas those with MSS tissues (*N* = 43) did not exhibit MMR gene pathogenic mutations. Thus, the PCR‐HRM method proficiently pinpoints tumors with verified germline MMR mutations, indicative of Lynch syndrome.

**Conclusion:**

Conclusively, the PCR‐HRM assay emerges as a swift and congruent diagnostic tool for microsatellite instability, boasting commendable sensitivity and specificity in colorectal cancer.

## Graphical abstract

The study demonstrated that the PCR‐HRM assay emerges as a swift and congruent diagnostic tool for MSI, boasting commendable sensitivity and specificity in colorectal cancer.

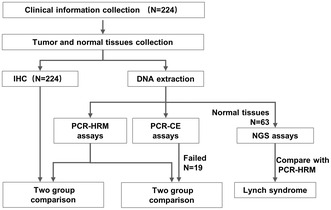

## 1 Introduction

Microsatellites comprise repetitive DNA sequences that are tandemly arranged in units of 1–6 nucleotides throughout the genome. The emergence of microsatellite instability (MSI) can be attributed to malfunctions in the mismatch repair (MMR) protein function, culminating in MSI. MSI tests are employed primarily to screen for Lynch syndrome, particularly in cases where colorectal cancer (CRC) manifests at an early age [1]. For stage‐II CRC, patients with MSI‐positive tumors generally exhibit a more favorable prognosis and might not derive significant benefits from fluorouracil‐centric chemotherapy [2]. For advanced CRC and other solid tumors, MSI serves as a potent biomarker, forecasting the responsiveness to PD‐1 immune checkpoint blockades (ICBs) [3]. Hence, ensuring the precise validation of MSI status remains imperative for the effective diagnosis and therapeutic interventions for patients with CRC.

MSI statuses are predominantly ascertained using the immunohistochemistry (IHC) methodology, which leverages specific antibodies targeting MMR genes (*MLH1*, *MSH2*, *MSH6*, and *PMS2*). Concurrently, polymerase chain reaction‐based capillary electrophoresis (PCR‐CE) techniques have gained traction. This method, characterized by multiplex fluorescent PCR‐CE, operates by detecting five mononucleotide repeats (BAT‐25, BAT‐26, NR‐21, NR‐24, and MONO‐27). Both the IHC and PCR‐CE assays stand as standard procedures endorsed for mismatch repair deficiency (dMMR) or MSI‐H testing, respectively [4].

Furthermore, novel molecular‐based assays have emerged for MSI detection, such as droplet digital PCR, real‐time PCR [5, 6], and next‐generation sequencing (NGS)–based methodologies. Typically, the NGS approach employs a custom panel encompassing hundreds of microsatellite loci, although this incurs significant costs [7, 8]. Although some NGS sequencing approaches can include additional genetic alterations, the detection requires robust computational algorithms for the analysis of NGS microsatellite data [9]. Furthermore, the operations in the wet lab are complex and the duration is relatively longer.

The IHC method indirectly determines the MSI status by evaluating the presence of MMR proteins. In contrast, PCR‐CE methods necessitate both tumor and nontumor tissues to scrutinize MSI genes. Stemming from these considerations, a real‐time PCR amplicon melting assay (PCR‐HRM) was developed to ascertain MSI status by solely examining tumor tissues. In a very recent study [10], the same PCR‐HRM‐based method for MSI detecting was developed by analyzing the tissues of 224 patients with CRC. Firstly, the dMMR mutation signature was identified by sequencing a panel of 725 microsatellite loci, and 13 MSI loci were screened by machine learning and laboratory testing. At last, eight distinct MSI loci including *ACVR2A*, *CENPQ*, *DIDO1*, *LRIG2*, *MRE11*, *PSIP1*, *SLC22A9*, and *TGFBR2* were combined to construct the PCR‐HRM assay. This innovative PCR‐HRM methodology can accurately determine a patient's MSI status from tumor tissue samples within 1.5 h, achieving 100% sensitivity and specificity. However, the sample size used in the initial development of this method was limited. To further validate the PCR‐HRM assay, our center conducted a verification study using samples from our own institutional cohort. In this study, we meticulously assessed the efficacy of the PCR‐HRM 8‐loci assay in discerning MSI status within CRC tumor samples and among Lynch syndrome patients. The IHC and the Promega MSI Analysis System served as our benchmark reference methodologies.

## 2 Materials and Methods

### 2.1 Tissue Sample Collection

**FIGURE 1:**
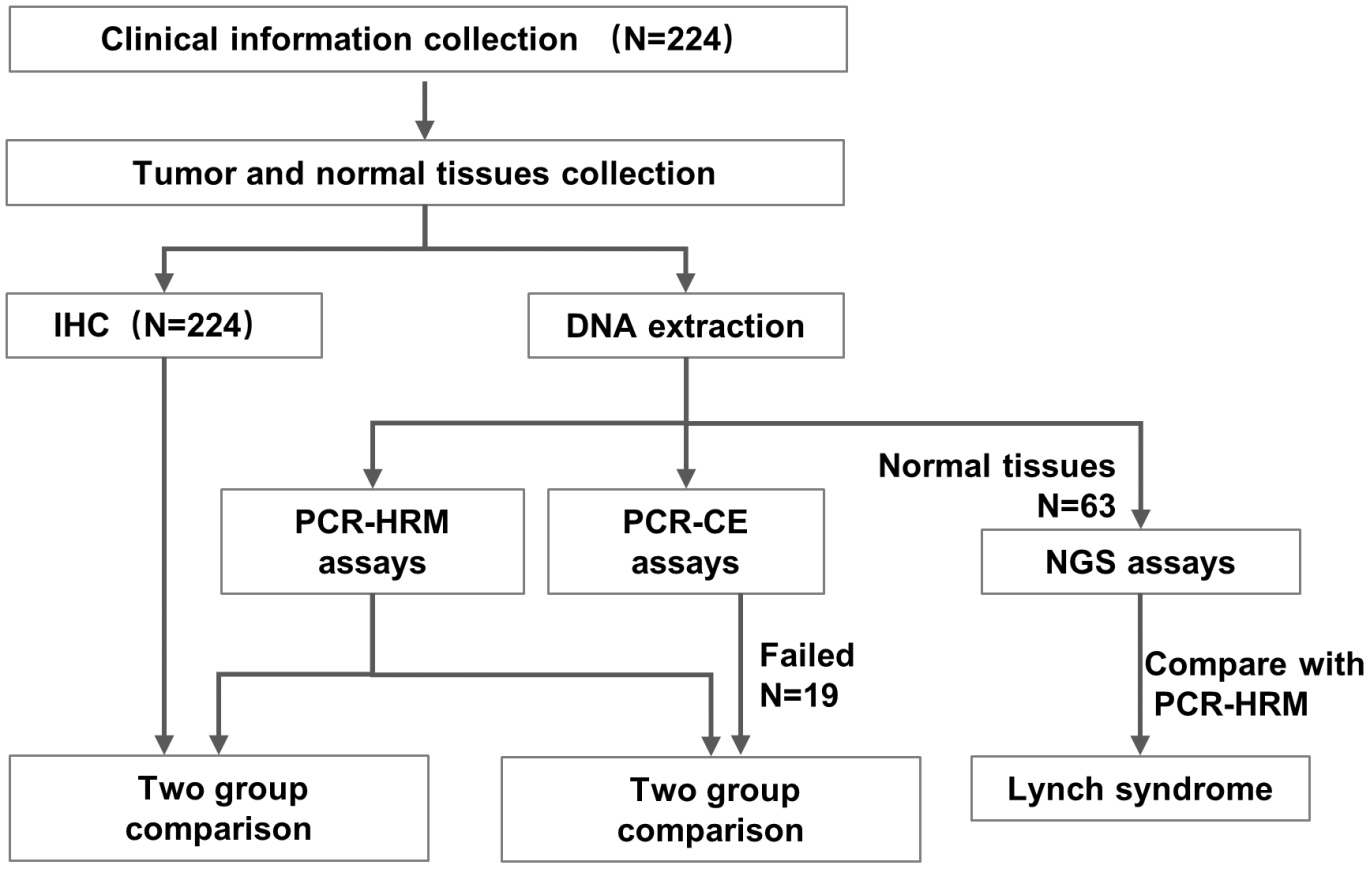
Process of patient enrollment and study design.

The participants included 242 patients with CRC who underwent surgical procedures at the Second Affiliated Hospital of Zhejiang University of Medicine (Zhejiang, China) between 2019 and 2022. The patient enrollment process was shown in Figure 1. Cases were deliberately chosen to enrich the representation of MSI‐H/dMMR cases, aiming for approximately a 50% ratio. This study secured approval from the local Ethics Committees of the Second Affiliated Hospital of Zhejiang University of Medicine (#IR2022070). Written informed consent was derived from the participants. Initially, the tumor cell content in the FFPE blocks was appraised using HE staining. FFPE sections with a tumor cell content exceeding 30% were consecutively retrieved from the block. Corresponding normal tissues from the tumor were also amassed for the Promega MSI assay. These sections served as the basis for either IHC detection or DNA extraction.

### 2.2 MMR Immunohistochemical (IHC) Assay

IHC evaluations for MLH1, MSH2, MSH6, and PMS2 were executed on FFPE tissue samples utilizing the PowerVision Two‐Step Histostaining Reagent. Specifically, 4‐μm FFPE sections underwent IHC procedures. The Anti‐MLH1 (M1) monoclonal antibody (J21119, Roche), Anti‐PMS2 (A16‐4) monoclonal antibody (J17928, Roche), Anti‐MSH2 (G219‐1129) monoclonal antibody (J22110, Roche), and Anti‐MSH6 (SP93) monoclonal antibody (J17390, Roche) were employed to determine the MMR status, strictly adhering to the manufacturer's guidelines. MMR‐deficient tumors displayed an absence of nuclear staining, or one or more markers exhibited nuclear staining in fewer than 10% of aggressive tumor cells. Tumors were designated as pMMR when nuclear staining was evident in a minimum of 10% of invasive tumor cells for all four markers.

### 2.3 DNA Extraction and PCR‐CE MSI Assay

We collected five slides each from tumor and matched nontumor 4‐μm FFPE sections. DNA was isolated using the Maxwell CSC DNA FFPE Kit, following the manufacturer's guidelines. This extracted DNA from both tumor and normal tissues served as the basis for MSI status analysis via PCR‐CE methods. We utilized the ProDx MSI Analysis System (ProDx MSI) for the PCR‐CE MSI assessment, adhering to the provided protocol. The ProDx MSI system comprises eight mononucleotide repeat markers (BAT‐52, BAT‐56, BAT‐59, BAT‐60, NR‐21, BAT‐25, BAT‐26, and MONO‐27) along with two pentanucleotide repeats.

### 2.4 PCR‐HRM MSI Assay

The novel PCR‐HRM MSI assay was developed by the Wondfo Biotech (Guangzhou, China). Briefly, DNA extracted from tumor samples was subjected to PCR amplification, followed by scoring via high‐resolution melt analysis, as directed (Wondfo). The setting of classification threshold was evaluated in detail in the process of product development. Compared with the gold standard IHC, the number of positive sites was determined by receiver characteristic (ROC) curve analysis and Jorden index, and the threshold was determined to be 2. The PCR process was conducted using the SLAN‐96S fluorescent quantitative PCR instrument (Hongshi, Shanghai, China). Samples displaying mutations in two or more markers were classified as microsatellite instability high (MSI‐H). In contrast, tissues presenting mutations in just one locus or devoid of mutations across all loci were designated as MSS. The entire duration, spanning FFPE sample preparation to MSI status reporting through the PCR‐HRM assay, approximates 90 min. This duration encompasses nucleic acid extraction from FFPE samples, PCR amplification, and high‐resolution melting detection analysis.

### 2.5 Procedures

1. A panel including 725 candidate microsatellite loci were screened from the public database. Ninety‐three paired tumor and matched paracancerous tissue samples were sequenced using Unique Molecular Identifier sequencing (UMI‐Seq). In total, 225 microsatellite loci that are specific to tumor and dMMR tissues were selected.
2. Thirty‐two MSI loci showed significantly difference in abundance were identified by K‐means clustering.
3. Thirteen MSI loci were identified in experimental conditions.
4. On the basis of the PCR results from 197 samples, we randomly selected 2, 3, 4, 5, 6, 7, 8, and 9 loci from the 13 potential loci, constructing 78, 286, 715, 1287, 1716, 1716, 1287, and 715 distinct combinations, respectively. Tissues were classified as MSI‐H if instability was observed in two or more of the candidate mononucleotide repeat loci. At a specificity of 100%, the 8‐loci combination produced the highest sensitivity (96.53%).
5. To assess the performance of the tumor MSI model encompassing 8 loci, tissues from 32 patients were applied for validation. When compared to the dMMR or pMMR results reported by pathologists, the 8‐loci MSI model could identify dMMR tumors with a specificity, sensitivity, positive predictive value, and negative predictive value all at 100%. In contrast, when compared to the IHC results, the Promega panel exhibited a specificity of 100% and a sensitivity of 93.75%.

### 2.6 NGS

DNA derived from normal tissue samples served the purpose of NGS. DNA libraries, inclusive of MLH1, MSH2, PMS2, and MSH6, were structured and sequenced on the Illumina NovaSeq6000 PE150 platform, achieving an average sequencing depth surpassing 10,000×. Sequence alignment was conducted against the human reference genome (GRCh37/hg19) using the Burrows‐Wheeler Aligner (BWA, v0.7.17) software, and variant annotations were executed using the Annovar (v201804) software. Variant pathogenicity was ascertained in alignment with the American College of Medical Genetics and Genomics (ACMG) guidelines.

### 2.7 Statistical Analysis

In this analysis, IHC results were designated as the gold standard for determining MMR status. The concordance between the PCR‐HRM assay and the other techniques, namely IHC and PCR‐CE assay, was assessed based on the overall diagnostic agreement. The formulas used for calculating sensitivity, specificity, and concordance were as follows:

- Sensitivity = true positives/(true positives + false negatives).
- Specificity = true negatives/(true negatives + false positives).
- Concordance = (true positives + true negatives)/total samples.

## 3 Results

### 3.1 Patients' Characteristics

The primary characteristics of the study subjects are detailed in Table 1. Of the 224 patients enrolled, 108 were males (48.21%) and 116 were females (51.79%). The age of the participants ranged between 15 and 92 years, with a median age of 63.07 years. The predominant staging among the patients was T3 (66.96%), followed by T4 and T2 both at 13.39%, and T1 at 4.02%.

**TABLE 1:** Patient characteristics.

Variables		*N*	%
Age (median)		63.07 (15–92)	
Gender	Male	108	48.21
	Female	116	51.79
MMR	dMMR	110	49.11
	pMMR	114	50.89
Tumor budding	Positive	62	27.68
	Negative	162	72.32
T stage	T1	9	4.02
	T2	35	15.63
	T3	150	66.96
	T4	30	13.39
N stage	N0	143	63.84
	N1	56	25.00
	N2	25	11.16
PCR‐HRM	MSI‐H	107	47.77
	MSS/MSI‐L	117	52.23
PCR‐CE	MSI‐H	96	42.86
	MSS/MSI‐L	109	48.66
	Not detected	19	8.48
MMR status	MLH1 negative	78	34.82
	PMS2 negative	84	37.5
	MSH2 negative	22	9.82
	MSH6 negative	25	11.16

A near even distribution was observed in the tissue types, with 49.11% (*N* = 110) of the patients having dMMR tissues, and 50.89% (*N* = 114) with pMMR tissues. MSI‐H was identified in 96 patients (42.86%) through PCR‐CE, whereas 105 patients were classified as MSS. The PCR‐CE assay encountered an 8.48% failure rate, with 19 patients being unevaluated due to inadequate DNA quality. Meanwhile, the PCR‐HRM assay detected MSI‐H tissues in 107 patients (47.77%) and MSS tissues in 117 patients (52.23%).

### 3.2 Concordance Between PCR‐HRM Assay and IHC

Upon employing the PCR‐HRM assay, results indicated 107 MSI‐H samples and 117 MSS/MSI‐L samples. Conversely, the IHC assay identified 110 dMMR tissues and 114 pMMR tissues. Detailed in Table 2, the PCR‐HRM assay successfully pinpointed 106 MSI‐H tissues from the 110 dMMR tissues and marked 113 MSS tissues from the 114 pMMR tissues. In juxtaposition with the IHC results, the sensitivity of the PCR‐HRM in identifying MSI‐H reached 96.36%, whereas its specificity stood at 99.12%. Thus, the overall concordance between the PCR‐HRM and IHC methods amounted to 97.77%.

**TABLE 2:** Sensitivity and specificity analysis of PCR‐HRM assays when compared with the IHC assay, PCE‐CE assay, and DNA sequencing results.

		PCR‐HRM		Total	Sensitivity [95% CI]	Specificity [95% CI]	Consistency [95% CI]
		MSI‐H	MSI‐L/MSS				
IHC	dMMR	106	4	110	96.36% [91.02–98.58]	99.12% [95.20–99.84]	97.77% [94.88–99.04]
	pMMR	1	113	114			
	Total	107	117	224			
PCR‐CE	MSI‐H	96	0	96	100% [96.15–100.00]	100% [96.60–100.00]	100% [98.16–100.00]
	MSI‐L/MSS	0	109	109			
	Total	96	109				
DNA sequencing	Pathogenic mutations	14	0	14	100% [78.47–100.00]	87.76% [75.76–94.27]	90.48% [80.74–95.56]
	No pathogenic mutations	6	43	49			
	Total	20	43				

**FIGURE 2:**
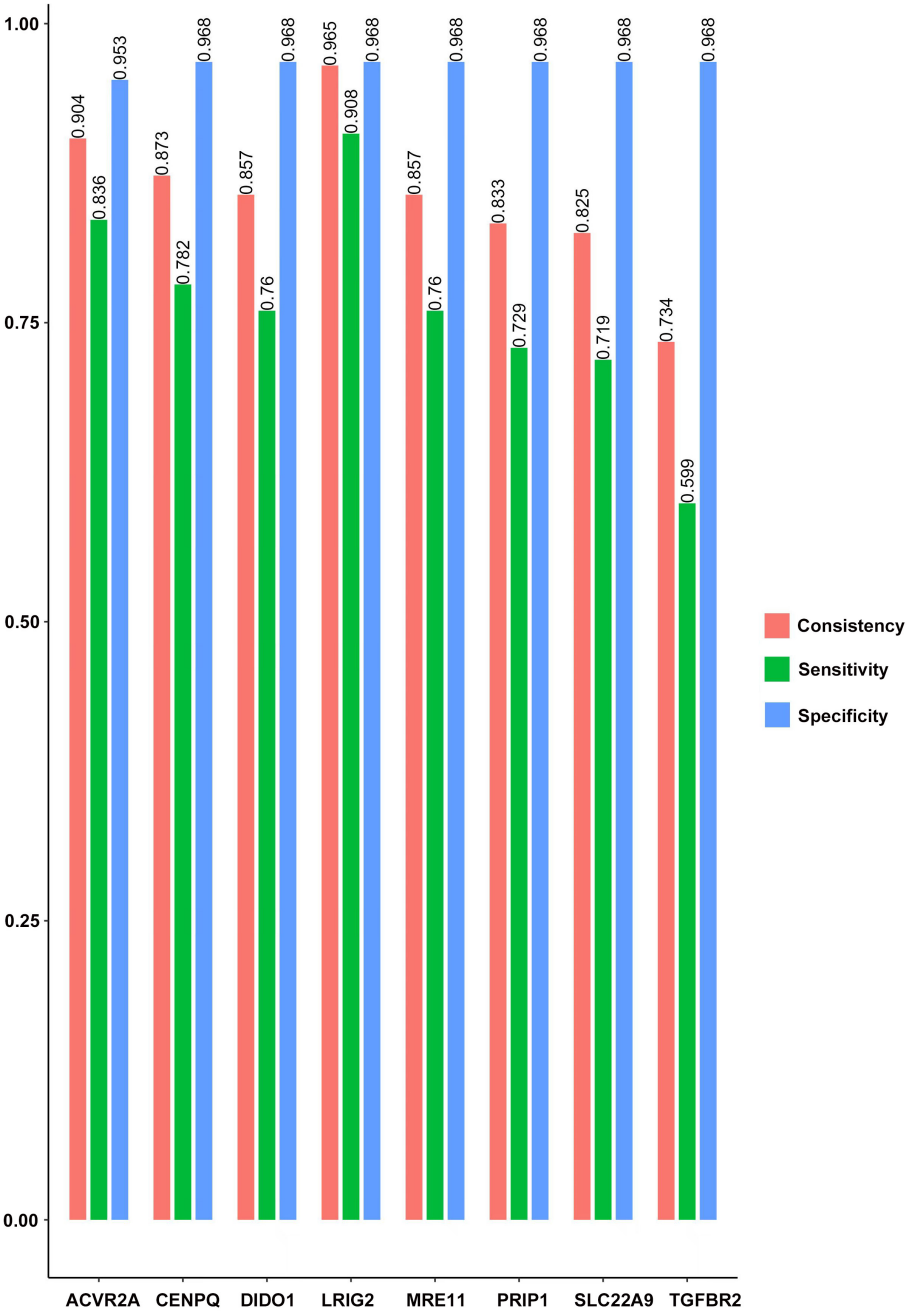
Consistency, sensitivity, and specificity of eight microsatellite instability markers when compared with the IHC results.

The PCR‐HRM approach analyzes eight specific MSI loci. As demonstrated in Figure 2, the sensitivity of these loci in detecting MSI‐H tissues varied, ranging from 66.36% to 92.73%, with LRIG2 presenting the highest sensitivity at 92.73%. Concerning specificity, all eight loci exhibited values >98%. ACVR2A was an exception with a specificity of 98.25%, whereas the remaining seven loci matched a specificity of 99.12%.

Delving further, as showcased in Table 3, of the 117 MSS tissues, a majority (*N* = 116) displayed no mutations whereas only one manifested a single mutation. On the contrary, in MSI‐H tissues, a substantial number exhibited more than 6 mutations. Specifically, 22 samples displayed 6 mutations, 39 had 7, and 32 harbored 8 mutations. Notably, mutations spanning 2, 3, 4, and 5 loci were observed in 2, 1, 4, and 7 samples, respectively.

**TABLE 3:** Number of mutant markers of PCR‐HRM 8‐loci assay.

MSI status	Number of samples	Number of mutant markers	Number of samples
MSI‐L/MSS	117	0	116
		1	1
MSI‐H	107	2	2
		3	1
		4	4
		5	7
		6	22
		7	39
		8	32

### 3.3 Concordance Between PCR‐HRM Assay and PCR‐CE Assay

To ascertain the MSI status of the specimens, the PCR‐CE assay was employed, which analyzed both tumor tissues and their adjacent paracancerous counterparts. A total of 19 samples were omitted due to inadequate DNA samples. As elucidated in Table 2, of the remaining 205 samples, the PCR‐CE assay classified 96 as MSI‐H and 109 as MSS/MSI‐L. Notably, the concordance between the PCR‐HRM and PCR‐CE methodologies was impeccable at 100% (with a confidence interval of 98.16%–100.00%). Both techniques unanimously classified 96 samples as MSI‐H and 109 as MSS/MSI‐L.

### 3.4 PCR‐HRM Assays Identified All Patients With Germline Pathogenic Mutations in MMR Genes

Exploring the potential of PCR‐HRM as a screening tool for Lynch syndrome, we enrolled 63 patients for high‐throughput sequencing to detect mutations in the MLH1, MSH2, PMS2, and MSH6 genes. Comprehensive data for each patient can be referenced in Table 2. Remarkably, of these, 14 patients harbored pathogenic mutations in at least one of the four MMR genes. Notably, PCR‐HRM identified all these patients' tumor tissues as MSI‐H. In contrast, 43 patients, whose tumors were determined to be MSI‐L/MSS by PCR‐HRM, did not present with any pathogenic mutations in the MMR genes. The comparative sensitivity and specificity between the two methods stood at 100% and 87.76%, respectively, resulting in an overall consistency of 90.48%. Thus, the PCR‐HRM method effectively screened all CRC patients with pathogenic mutations in this cohort. Furthermore, out of the 49 patients devoid of pathogenic mutations, six were classified as MSI‐H by PCR‐HRM. This points to the assay's ability to detect sporadic CRC cases.

## 4 Discussion

For CRC, the importance of the MSI test in guiding adjuvant chemotherapy and immunotherapy decisions is growing [11]. Accurate determination of MSI status in tumor tissues is paramount. In a recent paper, an 8 microsatellites loci‐based PCR‐HRM assay was reported for ascertaining the MSI status of CRC tumor tissues [10]. In this study, the effectiveness of this method was evaluated in determining MSI status. The PCR‐HRM MSI assay showed a high level of agreement with both IHC and PCR‐CE tests, demonstrating a 97.77% concordance with IHC and a 97.56% concordance with the PCR‐CE assay.

Of the 224 cases assessed, the PCR‐HRM method classified 107 patients with MSI‐H tissues and 117 with MSS/MSI‐L tissues. Notably, four dMMR tissues were identified as MSI‐L/MSS using the PCR‐HRM method. Compared with IHC results, the PCR‐HRM demonstrated a sensitivity and specificity of 96.36% and 99.12%, respectively. Furthermore, the ProDX MSI assay, rooted in the PCR‐CE method, has been shown to enhance the detection sensitivity for MSI‐High in CRC samples [9]. In our study, the sensitivity, specificity, and concordance rates were unanimously 100% between PCR‐HRM and ProDX MSI methods, underscoring the dependability of the PCR‐HRM technique in detecting MSI‐H.

**FIGURE 3:**
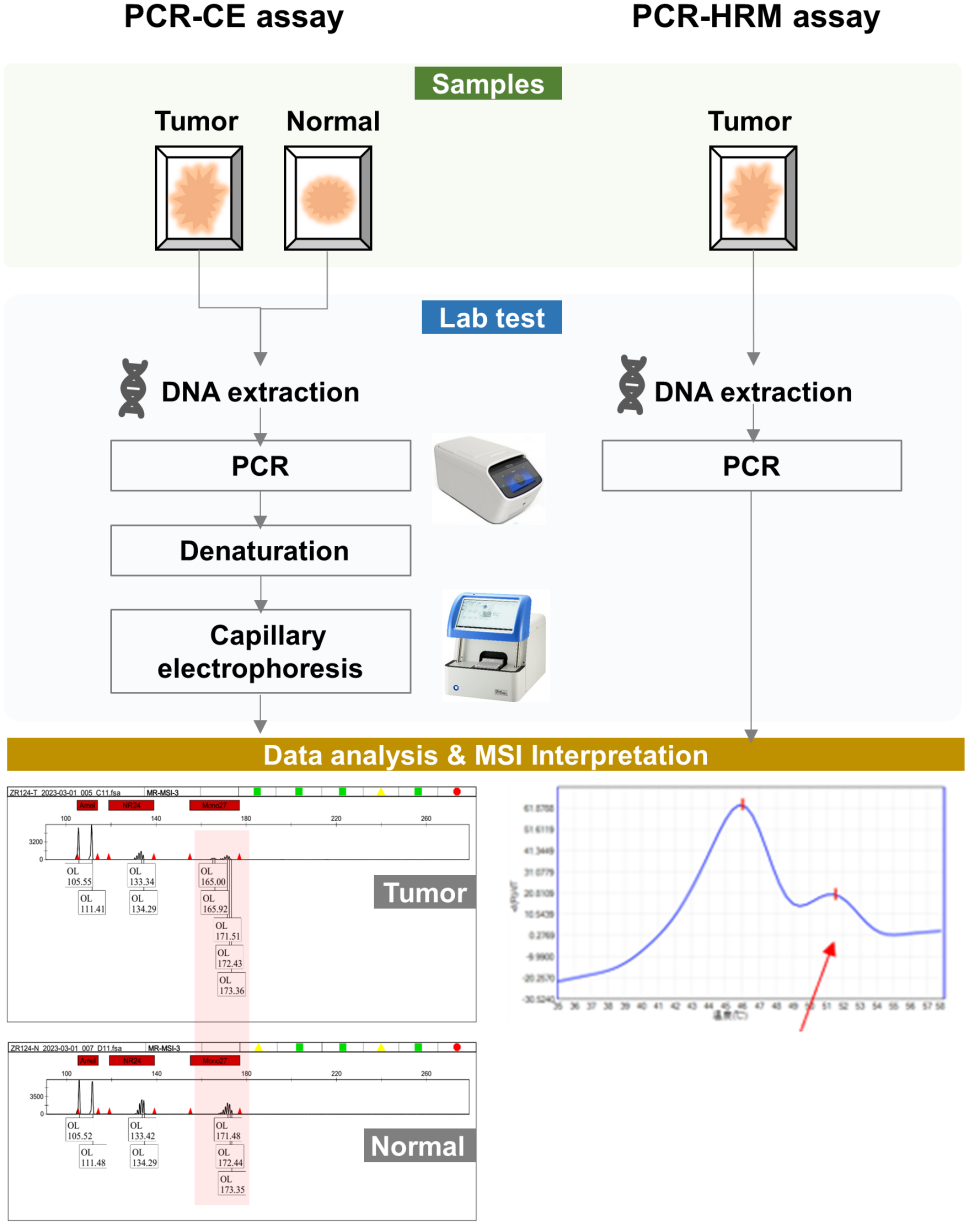
Comparison of the PCR‐CE and PCR‐HRM assays in the workflow and MSI interpretation. The comprehensive comparison was conducted across three key domains: sample collection, laboratory testing, and result analysis. The PCR‐CE methodology entails the collection of tumor and normal samples, subsequent to which they are subjected to PCR amplification and flow cytometry analysis. Result interpretation hinges upon the examination of locus‐specific peaks discerned between tumor and normal samples. In contrast, PCR‐HRM analysis solely demands tumor tissue, with result determination predicated on the melting curve analysis subsequent to qPCR.

In the PCR‐HRM assay, mutations were observed to be approximately equally distributed across the eight microsatellite loci (Figure 3). Apart from a single case, no mutations were detected in the eight microsatellites within all MSS/MSI‐L tissues. Conversely, most MSI‐H tissues exhibited mutations in at least six of the microsatellites (Table 3). Consequently, the majority of patients with MSI‐H tumors identified by PCR‐based methods presented with multiple microsatellite mutations, which could enhance the precision in screening patients with MSI‐H tissues.

Previous studies have indicated a notable discordance between IHC and molecular tests, especially in MSH6‐ and PMS2‐deficient endometrial carcinomas [12, 13]. In this study, we also observed the loss of expression of PMS2 and MLH1 in three cases that displayed discrepant MSI statuses between IHC and PCR‐HRM results. The discordance may due to loss of function missense mutations that do not affect epitope binding.

MSI‐H is present in roughly 15% of patients with CRC [14], of which about 3% are associated with Lynch syndrome [15]. Traditionally, MSI screening in colorectal and endometrial cancers serves as a diagnostic tool for Lynch syndrome due to its clinical significance. In our investigation, we assessed the efficacy of the PCR‐HRM method in pinpointing patients with germline MMR gene mutations. Specifically, mutations in the MMR genes MLH1, MSH2, MSH6, and PMS2 were examined via NGS in 63 patients, given that germline mutations in these genes predominantly contribute to deficient mismatch repair [16]. Comparing the outcomes of PCR‐HRM and NGS, patients (*N* = 14) with germline pathogenic mutations in MMR genes were also verified to possess MSI‐H tissues. In contrast, among the 43 patients with MSI‐L/MSS tissues, none exhibited mutations in the Lynch syndrome‐associated genes. This underscores the PCR‐HRM assay's potential in accurately identifying patients with pathogenic mutations in MMR genes, making it a promising tool for screening Lynch syndrome candidates.

IHC is commonly utilized to analyze MSI status in clinical practice. However, the evaluation of IHC can be subjective, contingent upon the expertise of the pathologist. PCR‐based tests and NGS can serve complementary roles, particularly when: (1) IHC results are unattainable, (2) there is a discrepancy between IHC results and the patient's familial clinical history, and (3) PCR‐based tests are used as companion diagnostic assays for immunotherapy in clinical trials. For instance, the FDA approved the Foundation One CDx as a companion diagnostic for Keytruda to pinpoint patients with MSI‐H solid tumors [17]. Within our study, one pMMR case was designated as MSI‐H through both PCR‐HRM and PCR‐CE, revealing seven mutations within the eight microsatellite loci upon using PCR‐HRM. Consequently, this tissue may be MSI‐H. Thus, integrating PCR and IHC methods might enhance the identification rate of patients with MSI‐H tumors.

Currently, the PCR‐CE method has found applications in clinical practice, with multiple kits, such as the Promega MSI Analysis System [18], receiving FDA approval. When juxtaposing the results from PCR‐HRM and PCR‐CE, the MSI status across all samples aligned consistently. Additionally, PCR‐HRM offers several advantages over PCR‐CE, IHC, and NGS techniques (Table 4). Firstly, for the types and quantity of samples, the PCR‐CE MSI assay demands nontumoral tissue, PCR‐HRM requires only tumor samples. As illustrated in Figure 3, DNA extracted from both tumor and normal tissues is amplified through PCR and subsequently analyzed via CE. MSI status is inferred by contrasting the outcomes from tumor and normal tissues. Conversely, PCR‐HRM provides MSI results upon completion of real‐time PCR, relying solely on tumor tissue. Given technical constraints, PCR‐based methods necessitate minimal DNA input than NGS, thereby requiring fewer tumor samples. For PCR‐HRM, roughly four slides of FFPE sections suffice for DNA extraction, whereas NGS demands a minimum of 10 slides. Secondly, the PCR‐HRM reduces both turnaround time and costs. On the basis of our work experience in the laboratory, the PCR‐HRM assay delivers dependable outcomes in approximately 1.5 h by automatic annotations and streamlined technology, contrast to 5 h for PCR‐CE [6], 2 weeks for NGS, and 1–2 days for IHC. Furthermore, PCR‐based techniques assess fewer than 10 microsatellite loci, in contrast to the hundreds examined by NGS. For the primers used in the experiment alone, the cost is lower in PCR‐based method. In addition, the operation in wet lab is simple for the PCR‐based method. Because of the characteristics of MSI loci, the capture and sequence for NGS are more challenging compared with nonrepetitive sequences. Furthermore, the MSI sequencing is highly susceptible to various factors during experimental processes, so the library construction, parameter adjustments, and bioinformatics analyses are more complex than the nonrepetitive sequences. For IHC, despite its routine clinical application, result interpretation is susceptible to a pathologist's subjectivity, emphasizing the need for expert judgment. In summary, considering factors such as the quantity of DNA input, the need for normal control samples, the complexity of experimental procedures, the ease of result interpretation, among others, the PCR‐CE method offers several advantages including short processing time, minimal sample requirements, simple experimental procedures, easy result interpretation, and rapid access to test results. It is a suitable detection method for application in clinical laboratories.

**TABLE 4:** Comparison of the technical performance between PCR‐HRM, NGS, and PCR‐CE assays.

Detection target	PCR‐HRM	PCR‐CE	NGS	IHC
Detection period	1.5 h	5 h	1–2 weeks	1–2 days
DNA input	80 ng	5–250 ng	50–250 ng	/
Test targets	8 loci	5–10 loci	Hundreds of loci	Mismatch repair proteins
Normal tissue as control	No	Yes	Yes or No	No
Tumor content	>10%	>20%	>10%	/
FFPE sections (4–5 μm)	4–6	1–10	10–15	4
Accuracy	High	High	High	Subjectivity
Experimental and interpretation	Simple	Medium	Complex	Complex
Economic cost	Low	Medium	High	Low

## 5 Conclusions

The PCR‐HRM MSI assay has consistently matched results obtained from IHC or PCR‐CE tests, eliminating the need for normal tissue and ensuring a swift turnaround. Moreover, the PCR‐HRM method proficiently identifies tumors with verified germline MMR mutations, which are indicative of Lynch syndrome.

## Author Contributions

Xueping Xiang, Xiaojing Ma, and Linlin Ying conducted the experiments, analyzed the data, and participated in manuscript writing. Hong Zou, the corresponding author, conceived and supervised the study, designed the experiments, and provided critical guidance throughout the research process. All authors reviewed and approved the final manuscript.

## Ethics Statement

This study involved human participants and was reviewed and approved by the local ethics committee of the Second Affiliated Hospital of Zhejiang Medical University (approval number: IR2022070). All procedures were conducted in accordance with the ethical standards set forth in the Declaration of Helsinki.

## Conflicts of Interest

The authors declare no conflicts of interest.

## Data Availability

The data that support the findings of this study are available from the corresponding author upon reasonable request.
